# Editorial to the Special Issue—“Recent Advances in Self-Assembled Peptides”

**DOI:** 10.3390/molecules24173089

**Published:** 2019-08-26

**Authors:** He Dong

**Affiliations:** Department of Chemistry & Biochemistry, The University of Texas at Arlington, Arlington, TX 76019, USA; he.dong@uta.edu

Peptide self-assembly is an interdisciplinary research area involving chemistry, life science, and materials science. Peptides, which can be either *de novo* designed or derived from natural protein sequences, are an important class of molecular building blocks to fabricate supramolecular nanostructures. Peptide self-assembly offers unique advantages in terms of control over the internal order and molecular packing of the building units. This has been proven to be an important factor in designing nanomaterials able to effectively interface with biological and/or non-biological targets to manipulate various chemical and biological processes in a controlled and predictable manner. Undoubtedly, supramolecular peptide assembly will continue to play an important role in the area of supramolecular chemistry and materials design to meet various needs for nanotechnological and biotechnological applications. This special issue aims to highlight some of the advances in both fundamental and practical aspects in the field of peptide self-assembly.

The review by Hudalla et al. highlighted new strategies for creating and functionalizing self-assembling peptide nanofiber scaffolds with sophisticated biomolecular ligands such as carbohydrates and recombinant proteins [1]. Conjugation of these ligands to a self-assembling peptide allows the fabrication of supramolecular nanostructures with controlled ligand type, density and multivalency, which are important factors to mediate cell surface receptor interactions. These exciting findings open up enormous opportunities to develop novel supramolecular biomaterials with highly tailored nanoarchitecture and unprecedented biological functionalities.

Nilsson et al. reported a significant discovery on the co-assembly of enantiomeric mixture of amyloid-β (16–22) into “rippled” β-sheet nanofibers, rather than “pleated” β-sheets as commonly observed for the self-assembly of enantiomeric pure peptides [2]. The intermolecular packing of the rippled β-sheets was elucidated using isotope-edited IR and solid-state nuclear magnetic resonance (NMR) spectroscopy, suggesting an alternating pattern of l- and d- peptide strands across the long fiber axis. The work provided fundamental insights into the molecular and supramolecular structure of rippled β-sheet nanofibers, as well as a general self-assembly route of enantiomeric β-sheet peptides.

Self-assembly of elastomeric peptides has great potential to generate biomaterials to mimic the property and function of native elastin protein scaffolds. Li et al. reported the self-assembly of amphiphilic peptides consisting of one or two VPGVG units derived from the hydrophobic domain on Tropoelastin [3]. Selected elastin-like peptides form supramolecular nanofibers and the supramolecular structures can be readily tuned by the overall hydrophobicity and temperature. This study demonstrates the great potential of developing thermal responsive self-assembling elastin-like peptides as functional cell scaffolds.

Peptide amphiphile micelles (PAMs) are a versatile nanostructured platform for targeted therapeutic delivery. Depending on the type of the hydrophobic domain, one can synthesize PAMs with different morphologies, such as spherical and cylindrical PAMs with potentially different biological properties. Chung et al. discovered that cylindrical PAMs that are modified by monocyte-targeting chemokine (MCP-1) have a greater ability to attract monocytes compared to their spherical counterparts [4]. The work has practical implications on controlling monocyte recruitment associated with cancer progression. The results also provide important guidance on designing PAMs with desired physical characteristic to suit specific needs for biological applications.

Self-assembling peptide hydrogels have shown great promise as an injectable multi-functional scaffold for drug delivery and tissue regeneration. Kumar et al. presented a nice summary of the current status of the progress and obstacle in the development and translation of peptide-hydrogel therapy in treating peripheral artery disease (PAD) [5]. The unmet medical needs can be potentially overcome by rational design of material scaffolds that allow for sustained angiogenic effect, *de novo* formation of microvasculature and neovasculature development.

Liquid–liquid phase separation (LLPS) is commonly observed in natural proteins, leading to non-fibrillar self-assembly. Considering many of the cell compartments are liquids that form by phase separation from the cytoplasm, the study of self-assembling peptides in association with LLPS has tremendous biological significance. Luo et al. reviewed the self-assembling process of Fused in Sarcoma (FUS), which is a DNA/RNA binding protein and in association with a variety of neurodegenerative diseases [6]. The review nicely summarizes both the aggregation and LLPS of FUS and their relationship with the pathology of diseases.

Polyelectrolyte complexes (PECs) are a result of liquid–liquid phase separation upon interaction between oppositely charged macromolecules. PECs from self-assembling peptides are an emerging area for the design of supramolecular nanomaterials. Leon et al. demonstrated a rational design approach to create PECs formed by an alternating sequenced d- and l-chiral patterns of charged and hydrophobic residues [7]. An important finding is that both the overall hydrophobicity and hydrophobic pattern play important roles on PECs formation and their properties. In addition to its fundamental significance with regard to PECs formation, rational design and patterning of the hydrophobic domain play key roles in effective encapsulation of a variety of therapeutic molecules with different hydrophobicity and charges.

Biophysical and biochemical assay are extremely crucial for the characterization and elucidation of the hierarchical structures of complex self-assembling systems. For example, self-assembled amyloid-β oligomers (AβOs) are implicated as neurotoxins to cause Alzheimer’s disease (AD) and the toxicity may be dependent on their quaternary structure. However, the polymorphic nature of AβO and their low abundance present significant challenges in accurate assessment of their structure–activity–function correlation. Doran et al. reported a simple yet effective biochemical assay to separate the distinct morphologies, quantify their relative abundance and ascertain their structures in comparison with a recombinant Aβ(M1-40) [8]. This method is highly promising to facilitate further functional characterization and biological activity of AβO and help establish a detailed understanding of AβO-mediated neurotoxicity.

These contributions aim to provide some of the current and future trends in the development of self-assembling peptide materials and effective tools to characterize these materials. We hope this special issue helps illustrate the fundamental design principle in self-assembling peptides and promote future research on the design, characterization, and translation of this technology to address unmet clinical needs.
